# Personality characteristics and competitive anxiety in individual and team athletes

**DOI:** 10.1371/journal.pone.0262486

**Published:** 2022-01-14

**Authors:** Supatcharin Kemarat, Apiluk Theanthong, Wichai Yeemin, Sutima Suwankan

**Affiliations:** Department of Sport Science and Sport Development, Faculty of Allied Health Sciences, Thammasat University, Rangsit, Pathumthani, Thailand; La Sapienza University of Rome, ITALY

## Abstract

The purposes of this study were to investigate differences in personality and competitive anxiety depending on types of sports and gender, and to determine the influence of personality on competitive anxiety. Participants included 237 athletes (134 men and 103 women) who participated in the Thailand University Games, 2020. They were classified as individual (n = 114) and team (n = 123) athletes. Personality characteristics and competitive anxiety were assessed by using NEO five-factor inventory and sport competitive anxiety test. Differences between individual and team athletes and between gender were tested by using independent t-test. Relationships between personality and competitive anxiety were analyzed by using Pearson product-moment correlation coefficient. Moreover, multiple regression analysis was used to measure the contributions of personality on competitive anxiety. The results showed that competitive anxiety was significant difference between individual and team athletes (p = 0.03, d = 0.28). However, there was no difference in personality between groups. When compared between gender, there were significant differences in competitive anxiety (p < 0.001, d = 0.52) and the agreeableness (p = 0.04, d = -0.26) component of personality between female and male athletes. From the correlation analyzes, four characteristics of personality showed significant associations with competitive anxiety including neuroticism (r = -0.472, ρ < 0.001), extraversion (r = 0.184, ρ = 0.005), agreeableness (r = 0.147, ρ = 0.024), and conscientiousness (r = 0.202, ρ = 0.002). Among five personality factors, the neuroticism had minimally negative effect on competitive anxiety (β = -0.52) with percentage of prediction of 22%. These can be concluded that types of sport and gender are the important factors affecting personality and competitive anxiety. The athletes with certain personality traits were more susceptible to competitive anxiety. Importantly, the neuroticism could serve as a prediction of the competitive anxiety in all collegiate athletes.

## Introduction

Anxiety is a negative emotional feeling that affect perceptions in sport competitions. Many athletes consider anxiety to be debilitative towards their motor abilities which may result in a decrease in performance [1]. Sport psychologists generally differentiate anxiety into trait anxiety which relates to a more stable aspect of personality and state anxiety that is temporary feelings in a particular situation. Both types of anxiety had negative correlation with sport performance [2]. Level of anxiety changes dramatically during competition as its cognitive and somatic components alter with time and situation [3]. The cognitive anxiety may show as “negative expectations, worries, and concerns about oneself, the situation at hand, and potential consequences” while the somatic anxiety refers to as “the perception of one’s physiological arousal”. With anxiety related problems, athletes feel threatened and try to deal with the issue themselves without constructive plan. Since competition require highly demands of success, athletes expect that effective control of anxiety could help achieving a successful result [4]. Khan et al. [5] stated that the anxiety affects the overall performance through physiological and behavioral effects and personality changes. Anxiety has physiological effects either directly or indirectly on body functions such as muscles shaking, fast heartbeat, sweating and fast breathing [5]. Anxiety affects individual’s feelings and perceptions that could induce behavioral changes such as anger, displeasure, problems in communication and unfriendliness. When athletes could not cope with anxiety, personality shall be changed and negatively effect on performance.

Personality describes the consistent patterns of thoughts, emotion, and behavior which characterize each person across time and situations. Thus, personality as the psychological qualities contribute to an individual’s enduring and distinctive patterns of feeling, thinking and behaving [6]. There are five components of personality including neuroticism, extraversion, openness to experience, agreeableness, and conscientiousness [7]. These could differentiate individual characters including hereditary, stable over time and across cultures [8]. With varying circumstances in competition, personality trait appeared to be associated with psychological characteristics of sportsmen [9–12]. Habib et al. [13] showed that the agreeableness, conscientiousness, and openness could be the significant predictors of sports performance among university athletes. Moreover, female soccer players who exhibited neuroticism and conscientiousness characters were more likely to be coachable athlete. Similarly, both male and female boxers with high level of personality traits apparently showed self-control and self-efficacy [11]. Furthermore, Petito et al. [14] found that the occurrence of anxiety and depressive symptoms existed with an increasing serotonin transporter which could define the neuroticism aspect of trait anxiety.

Previous researches have been studied personality characteristics in a variety of sports and found differences among types of sports [15, 16], gender [12, 17], race [18], skill level and experience [19]. Most studies have been investigated in the highly skilled athletes who were American or lived in Western countries. To date, information regarding personality and competitive anxiety in Asia collegiate athletes is scarce. In Thai collegiate athlete or University athletes, some athletes will become highly skilled players and capable of getting involved in the highest national tournament. Understanding their behavior and mental characteristics would be helpful in sport-related psychological management. Therefore, this study aimed at investigating personality and competitive anxiety among collegiate athletes and measure differences between types of sports and between gender. Moreover, the influence of personality on competitive anxiety was examined for all athletes and specifically in a particular type of sports.

## Materials and methods

### Participants

A total of 237 collegiate athletes (134 Males and 103 Females, age 18–25 years) who participated in the Thailand University Games, 2020 were recruited in this study. Among them, 114 athletes participated in the individual sports including track and field, board games, shooting, equestrian, jujitsu, swimming, fencing, golf, taekwondo, climbing, panchak silat, fit ball, karate-do, tennis, badminton, table tennis, and petanque. Another 123 athletes participated in the team sports involving electronic sport, football, futsal, volleyball, beach volleyball, sepak takraw, basketball, social dance, rowing, and rugby. All participants were the collegiate athletes and had participated in the Thailand University Games for almost 5 years (5.16 ± 3.15 years). Ethical approval was obtained prior to data collection from Ethics Review Sub-Committee for Research Involving Human Research Subjects of Thammasat University (number 094/2020).

### Measurements

Prior to the assessment, all participants provided written informed consent. Then, they provided information concerning personality and sport anxiety by completing the NEO five-factor inventory (NEO-FFI) and the sport competitive anxiety test (SCAT) questionnaires. Filling answers were carried out on the spot by using pencil-and-paper versions. Overall, participants took approximately 20 min to complete these questionnaires.

#### Personality characteristics

The NEO Five-Factor Inventory (NEO-FFI) [20] consists of 60 items self-descriptive statements that participants respond to using a 1 (*strongly disagree*) to 5 (*strongly agree*). The questionnaire measures five factors of personality. First, neuroticism refers to the vulnerability to emotional instability and self-consciousness, for example, “I am not a worrier” and “At times I have felt bitter and resentful”, etc. Second, openness to experience is characterized by the cognitive disposition to creativity and esthetics, for example, “I think it’ s interesting to learn and develop new hobbies” and “I have a lot of time intellectual curiously”, etc. Third, extraversion reflects the tendency to be gregarious, enthusiastic, assertive, and to seek excitement, for example, “I like to have a lot of people around me” and “I shy away from crowds of people”, etc. Fourth, agreeableness refers to the tendency to be warm, kind, gentle, trusting, and reliable, for example, “I try to be courteous to everyone I meet” “Some people think of me as cold and calculating”, etc. Last, conscientiousness is the tendency toward dutifulness and competence, for example, “I keep my belongings neat and clean” and “I never seem to be able to get organized”, etc. From the analyzes, all scales showed the internal consistency of 0.70. The Cronbach’s Alpha score ranged from the highest factor of 0.80 for Neuroticism, followed by 0.78, 0.77, and 0.74 for conscientiousness, extraversion, and agreeableness, respectively, and the lowest factor was 0.22 for openness to experience.

#### Sport competition anxiety

The sport competitive anxiety test (SCAT) consists of 15 items [21] including the first 10 items explaining symptoms associated with sport competition anxiety while the remaining 5 items were not scored but included to reduce the likelihood of an internal response-set bias. The participants were requested to respond that how do they usually feels during the competition, for example, “Before I compete, I feel uneasy” and “Before I compete, I am calm”, etc. Based on the participant’s feeling, each item was scored as hardly ever (1), sometimes (2), and often (3). The total scores were calculated and interpreted into three levels of anxiety including low (0–10), moderate (11–20), and high (21–30). The higher scores indicate the more risk of being anxious during the matches. From the analyzes, the internal consistency of all scales was 0.80.

### Statistical analysis

To verify the research problem, statistical analyses were performed using Open-Source Software R version 4.1 and R Studio. Descriptive statistics were analyzed by using mean, standard deviation, frequency, and percentage. Normality of all data were checked by z-value of the skewness and kurtosis. For medium-sized samples (50 < n < 300), z-value ± 3.29 was considered as normally distributed [22]. To investigate differences in personality characteristics and sport competitive anxiety, independent t-tests was used to compare between types of sport (individual and team sports) and between gender (male and female). Magnitude of the difference was assessed by using Cohen’s d [23] which interpreted as small (0.0 < d < 0.2), medium (0.3 < d < 0.5), and large (d > 0.6). A multiple regression analysis was used to estimate the relative contributions of independent variables (neuroticism, extraversion, openness to experience, agreeableness, and conscientiousness) on the competitive anxiety. Pearson’s correlation coefficient was used to verify which variable would be initially considered in these regression models. All variables met the criteria that r was less than 0.80 and variance inflation factor was less than 10 [24]. With magnitude of correlation, relationships between personality characteristics and competitive anxiety were interpreted as trivial (r ≤ 0.1), small (0.1 < r ≤ 0.3), moderate (0.3 < r ≤ 0.5), large (0.5 < r ≤ 0.7), very large (0.7 < r ≤ 0.9), and almost perfect (r > 0.9) [25]. Significant level was set as p-value less than 0.05.

## Results

For all 237 participants, 114 athletes (48.10%) and 123 athletes (51.90%) were classified as individual and team athletes. Comparisons between groups showed that there was significant difference in competitive anxiety, but no difference in personality was found between them (Table 1). The results showed that the individual athletes had significantly higher level of competitive anxiety than the team athletes with small effect (d = 0.28, p = 0.03). When compared between gender, there were significant differences in the competitive anxiety between female (22.40 ± 3.45) and male (20.49 ± 3.85) athletes with moderate effect (d = 0.52, p < 0.001). Moreover, the agreeableness component of personality was significant difference between gender (2.48 ± 0.54 and 2.62 ± 0.53, respectively) with small effect (d = -0.26, p = 0.04). For other personalities, both female and male athletes had similar characteristics.

**Table 1:** Comparisons of personality and competitive anxiety between individual and team athletes.

Variables	Individual (n = 114)	Team (n = 123)	Mean Difference	[95% CI]	t	d	*p*
	Mean (SD)	Mean (SD)					
1. Personality							
*Neuroticism*	3.20 (0.66)	3.13 (0.55)	0.06	[-0.09, 0.22]	0.79	0.1	0.43
*Extraversion*	2.69 (0.56)	2.69 (0.55)	-0.00	[-0.14, 0.14]	-0.03	-0.01	0.97
*Openness to experience*	2.99 (0.34)	2.93 (0.32)	0.06	[-0.02, 0.15]	1.44	0.19	0.15
*Agreeableness*	2.49 (0.54)	2.62 (0.53)	-0.12	[-0.26, 0.01]	-1.81	-0.24	0.07
*Conscientiousness*	2.55 (0.56)	2.58 (0.55)	-0.03	[-0.17, 0.11]	-0.44	-0.06	0.66
2. Competitive anxiety	21.88 (3.73)	20.80 (3.80)	1.08	[0.12, 2.04]	2.21	0.28	0.03*

SD = standard deviation; 95% CI = 95% confidence interval for mean difference;

t = t statistic value; p = significance level; d = effect size Cohen’s d;

*p < 0.05.

For the correlation analyzes (Table 2), there were significant relationships between four components of personality and competitive anxiety. Among these personalities, the neuroticism had moderate negative correlation with competitive anxiety (r = -0.472, p < 0.001) whereas the extraversion (r = 0.184, p = 0.005), the agreeableness (r = 0.147, p = 0.024), and the consciousness (r = 0.202, p = 0.002) showed small positive correlations with competitive anxiety.

**Table 2:** Relationships between personality and competitive anxiety in all athletes (n = 237).

Variables	2	3	4	5	6	Mean (SD.)
1. Neuroticism	-0.438***	0.008	-0.482***	-0.436***	-0.472***	3.16 (0.60)
2. Extraversion		-0.027	0.494***	0.641***	0.184**	2.69 (0.55)
3. Openness to experience			-0.079	0.050	-0.028	2.96 (0.33)
4. Agreeableness				0.543**	0.147*	2.56 (0.53)
5. Conscientiousness					0.202**	2.56 (0.55)
6. Competitive anxiety						21.32 (3.79)

SD = standard deviation;

* = p < 0.05,

**p < 0.01,

***p < 0.001.

Regarding multiple linear regression analysis (Table 3), all components of personality could explain the competitive anxiety with percentage of prediction of 22.00 for all athletes. Among five characteristics, the neuroticism was the only factor that had significantly negative effect on the competitive anxiety (β = -0.52, p < 0.001). Moreover, when considering in the individual athletes, the neuroticism showed significantly negative effect on the competitive anxiety (β = -0.55, p < 0.001). For the team athletes, the neuroticism (β = -0.56) and the agreeableness (β = 0.36) were significantly associated with the competitive anxiety (p < 0.001). Taken all five factors into the model could explain the total variances in the competitive anxiety of 29% and 22% for the individual and team athletes, respectively.

**Table 3:** Multiple linear regression analyzes in all athletes and different types of sport.

	Variables	B [95%CI]	Std. Error	β	t	*p*
**All Athletes (n = 237)**	Neuroticism	-3.24 [-4.09, -2.39]	0.43	-0.52	-7.51	0.00***
	Extraversion	-0.13 [-1.19, 0.93]	0.54	-0.02	-0.24	0.81
R = 0.23, Adjusted R^2^ = 0.22, F = 14.15, P < 0.001	Openness to experience	-0.43 [-1.75, 0.88]	0.67	-0.04	-0.65	0.52
	Agreeableness	-0.91 [-1.95, 0.12]	0.53	-0.13	-1.74	0.08
	Conscientiousness	0.42 [-0.67, 1.52]	0.56	0.06	0.76	0.45
	Constant	34.44 [-]	3.24	-	10.62	0.00***
**Individual Athletes (n = 114)**	Neuroticism	-3.12 [-4.16, -2.08]	0.52	-0.55	-5.95	0.00***
	Extraversion	-0.68 [-2.03, 0.67]	0.68	-0.10	-0.10	0.32
R = 0.32, Adjusted R^2^ = 0.29, F = 10.1, P < 0.001	Openness to experience	-0.58 [-2.31, 1.14]	0.87	-0.05	-0.67	0.50
	Agreeableness	1 [-0.39, 2.39]	0.70	0.14	1.43	0.16
	Conscientiousness	-0.22 [-1.65, 1.21]	0.72	-0.03	-0.31	0.76
	Constant	33.49 [-]	4.12	-	8.12	0.00***
**Team Athletes (n = 123)**	Neuroticism	-3.85 [-5.21, -2.48]	0.69	-0.56	-5.58	0.00***
	Extraversion	0.32 [-1.28, 1.91]	0.50	0.05	0.40	0.69
R = 0.25, Adjusted R^2^ = 0.22, F = 7.93, P < 0.001	Openness to experience	-5.58 [-2.49, 1.34]	0.97	-0.05	-0.60	0.55
	Agreeableness	-2.6 [-4.12, -1.07]	0.77	0.36	-3.67	0.00***
	Conscientiousness	0.73 [-0.89, 2.36]	0.82	-0.11	0.90	0.37
	Constant	38.58 [-]	4.88	-	7.90	0.00***

B = unstandardized coefficient; Std. Error = standard error of unstandardized coefficient;

β = standardized coefficient; t = t statistic value; 95% CI = 95% confidence for B,

*** = p < 0.001.

## Discussion

The main purposes of this study were to investigate changes in personality characteristics (neuroticism, extraversion, openness to experience, agreeableness, and conscientiousness) and competitive anxiety as a consequence of types of sport and gender. Furthermore, the influence of personality on competitive anxiety was examined for all athletes and for different types of sport (individual and team athletes).

### Comparisons of personality and anxiety between the individual and the team athletes

Our study found that none of the personality characteristics showed differences between individual and team athletes. In contrast to previous studies, variations in personality characteristics existed between types of sport [15, 26, 27]. Laborde et al. [27] reported that the individual athletes showed higher score on the neuroticism and the openness to experience which indicated positive characteristic of personality. With responsibility that comes from performing alone, individual athlete needs to possess greater enduring personal dispositions to achieve successful results. Interestingly, our results showed that the team athletes tended to experience higher scores of agreeableness as compared with those in the individual group (p = 0.07). The agreeableness is one of the personality traits that is characterized by cooperative, generous, and flexible behavior. Thus, playing with a team requires the agreeable teammate to promote positive group processes such as cohesion, cooperation, and conflict resolution. Regarding effect of gender, the present study found that the male athletes had significantly higher score in the agreeableness than the female athletes. This was not in line with previous studies in other cultures and nationalities [17, 28]. The higher level of the agreeableness were shown in Australian women [28] and Canadian female athletes [17]. This discrepancy may be due to cultural differences. For the current study, it was likely that Thai men tend to be open-minded, not overthinking, good relationship, and high commitment, so that resulted in high level of agreeableness accordingly. Thus, culture of origin or social roles might be the potential factor affecting personality trait of the athletes.

Regarding the competitive anxiety, our results showed that the individual athletes had slightly higher score than those in the team athletes. Similar results were also reported in collegiate players [5]. Since the anxiety level was not too high (20–22 scores), this may have slightly less effect on athlete’s performance. As stated in the inverted U theory, the peak performance could be achieved when level of pressure or arousal is appropriate for the sport performance. For the present study, these could be that the individual athletes had proper levels of anxiety that they could control and thus help facilitating superior performance accordingly.

Concerning gender differences, the present study found significantly higher score of competitive anxiety in women as compared with men. Similarly, Walton et al. [29] revealed that female athlete exhibited higher rates of mental health symptoms which possibly caused mental well-being problems and adverse life events. Additionally, female athlete may be more susceptible to external factor such as stress, drama, and fear, which usually occur during competition [30]. It has been generally accepted that emotional focused technique is one of the mental skill practice that could help individuals become more aware of their emotions and thus lesson adverse effects of unstable emotion. Thus, it is suggested that women should perform emotional-focused technique to effectively deal with factors related to competitive anxiety.

### Relationships between personality and competitive anxiety

In the present study, we found the significant relationships between four components of personality and competitive anxiety among all athletes. Among these personalities, the neuroticism was negatively associated with the competitive anxiety while the extraversion, the agreeableness, and the consciousness had positive correlations. Similarly, previous studies found the associations between these personalities and other psychological parameters such as self-efficacy, self-control [11, 14]. From our results, the openness to experience component of personality was not correlated with competitive anxiety. This was in line with Sabu and Thomas [31] in young adult. This may cause some negative effect since openness to experience could define the more willing to embrace new things, fresh ideas, and novel experiences.

From linear regression analyzes, our finding showed that the neuroticism could minimally effect on the competitive anxiety (β = -0.52). Similarly, a previous study in young taekwondo athletes found that the neuroticism could predict changes in psychophysiological parameters with percentage of prediction of 21% [32]. Neuroticism is the tendency to experience negative emotions, such as anger, anxiety, or depression, which can be referred to as emotional stability. So, it might be associated with anxiety in athletes who normally exposed to tremendous psychological stress. Moreover, there may be some possible determinants of anxiety among athletes, and the degree to which they may or may not have been experienced in sport tournament. It was possible that athletes may have sport specific demands such as overwhelming demands of training and psychological pressure or failure, which were related to higher levels of anxiety in elite athlete [14].

According to theory of personality [33, 34], the neuroticism is interlinked with low tolerance for stress or stimuli. Athletes who had high score in the neuroticism may be emotionally reactive and vulnerable to stress. Also, they may be more likely to interpret ordinary situations as life threatening as well as perceive minor frustrations as hopelessly difficult. Consequently, their negative emotional reactions tend to persist for unusually long periods of time. According to Petito et al. [14], personality showed correlation with serotonin transporter polymorphisms which characterized the occurrence of anxiety and depressive symptoms in elite athletes. So, it was possible that the neuroticism could mediate cognitive anxiety and emotional arousal control, and thus help predicting adverse mental health outcomes in athletes.

Nevertheless, our study has some limitations regarding measurement of personality characteristics. With Thai version of NEO Five-Factor Inventory, when considering factor loaded on the other factors, we observed that the openness to experience was less constant than other factors. So, interpretation for Thai may be differed from the original version in American culture. For example, “a thoughtful and will lead to creativity” may be misinterpreted as an instable or lazy. From the analyzes, the internal consistency in this parameter was quite low (0.23–0.79). This was also in line with a previous study in Thailand [35].

## Conclusions

Our main findings showed that the individual athletes had significantly higher level of competitive anxiety as compared with those in the team athletes. However, personality was similar in both groups. For the effect of gender, significantly differences in competitive anxiety and the agreeableness component of personality existed between women and men. Moreover, personality characteristics were significantly correlated with competitive anxiety. Among all personality characteristics, only the neuroticism factor showed minimally negative effect on competitive anxiety. All personality factors could predict changes in the competitive anxiety of 22% for all athletes and a group of team athletes, and 29% for the individual athletes. From our findings, we suggested that monitoring factors related to competitive anxiety and personality could help implementing early intervention approaches to reduce mental problems within the teams as well as to help athletes dealing with anxiety themselves.
